# PET/MRI of hypoxia and vascular function in ER-positive breast cancer: correlations with immunohistochemistry

**DOI:** 10.1007/s00330-023-09572-6

**Published:** 2023-05-11

**Authors:** Julia C. Carmona-Bozo, Roido Manavaki, Jodi L. Miller, Cara Brodie, Corradina Caracò, Ramona Woitek, Gabrielle C. Baxter, Martin J. Graves, Tim D. Fryer, Elena Provenzano, Fiona J. Gilbert

**Affiliations:** 1 Department of Radiology, School of Clinical Medicine, University of Cambridge, Box 218 – Cambridge Biomedical Campus, Cambridge, CB2 0QQ UK; 2 Cancer Research UK – Cambridge Institute, University of Cambridge, Li Ka Shing Centre, Robinson Way, Cambridge, CB2 0RE UK; 3 Wolfson Brain Imaging Centre, Department of Clinical Neurosciences, School of Clinical Medicine, University of Cambridge, Box 65 – Cambridge Biomedical Campus, Cambridge, CB2 0QQ UK; 4 Cambridge Breast Unit, Cambridge University Hospitals NHS Foundation Trust, Box 97 – Cambridge Biomedical Campus, Cambridge, CB2 0QQ UK

**Keywords:** Breast cancer, Hypoxia, Microvessel density, Carbonic anhydrase IX, PET/MRI

## Abstract

**Objectives:**

To explore the relationship between indices of hypoxia and vascular function from ^18^F-fluoromisonidazole ([^18^F]-FMISO)-PET/MRI with immunohistochemical markers of hypoxia and vascularity in oestrogen receptor–positive (ER +) breast cancer.

**Methods:**

Women aged  > 18 years with biopsy-confirmed, treatment-naïve primary ER + breast cancer underwent [^18^F]-FMISO-PET/MRI prior to surgery. Parameters of vascular function were derived from DCE-MRI using the extended Tofts model, whilst hypoxia was assessed using the [^18^F]-FMISO influx rate constant, *K*_i_. Histological tumour sections were stained with CD31, hypoxia-inducible factor (HIF)-1α, and carbonic anhydrase IX (CAIX). The number of tumour microvessels, median vessel diameter, and microvessel density (MVD) were obtained from CD31 immunohistochemistry. HIF-1α and CAIX expression were assessed using histoscores obtained by multiplying the percentage of positive cells stained by the staining intensity. Regression analysis was used to study associations between imaging and immunohistochemistry variables.

**Results:**

Of the lesions examined, 14/22 (64%) were ductal cancers, grade 2 or 3 (19/22; 86%), with 17/22 (77%) HER2-negative. [^18^F]-FMISO *K*_i_ associated negatively with vessel diameter (*p* = 0.03), MVD (*p* = 0.02), and CAIX expression (*p* = 0.002), whilst no significant relationships were found between DCE-MRI pharmacokinetic parameters and immunohistochemical variables. HIF-1α did not significantly associate with any PET/MR imaging indices.

**Conclusion:**

Hypoxia measured by [^18^F]-FMISO-PET was associated with increased CAIX expression, low MVD, and smaller vessel diameters in ER + breast cancer, further corroborating the link between inadequate vascularity and hypoxia in ER + breast cancer.

**Key Points:**

*• Hypoxia, measured by [* ^*18*^ *F]-FMISO-PET, was associated with low microvessel density and small vessel diameters, corroborating the link between inadequate vascularity and hypoxia in ER + breast cancer.*

*• Increased CAIX expression was associated with higher levels of hypoxia measured by [* ^*18*^ *F]-FMISO-PET.*

*• Morphologic and functional abnormalities of the tumour microvasculature are the major determinants of hypoxia in cancers and support the previously reported perfusion-driven character of hypoxia in breast carcinomas.*

**Supplementary Information:**

The online version contains supplementary material available at 10.1007/s00330-023-09572-6.

## Introduction

In breast cancer, like most solid tumours, the pathophysiology of the microenvironment is characterised by an irregular vascular network resulting in perfusion anomalies and hypoxia. The transcription factor hypoxia-inducible factor 1α (HIF-1α) is regarded as the master regulator of cellular adaptation to hypoxia supporting angiogenesis and the metabolic rewiring of tumours to a state which is less dependent on oxygen and nutrients [1]. Hypoxia is associated with tumour aggressiveness, therapeutic resistance, and metastasis in various cancers [2, 3], and is also recognised as a key factor contributing to poor clinical outcomes in patients with oestrogen receptor–positive (ER +) breast cancer [4]. Previous studies have demonstrated that overexpression of hypoxia-associated proteins at diminished oxygen levels, including HIF-1α and its downstream target, carbonic anhydrase IX (CAIX), is associated with suppressed oestrogen receptor-α (ER-α) levels [5, 6], a dedifferentiated phenotype [7], resistance to endocrine treatment [8–10], breast cancer recurrence [11], and shorter disease-free survival [12].

Whilst the potential clinical utility of HIF-1α and CAIX in ER + breast cancer has been discussed by several authors [7–12], the relationships between the in vivo tumour pathophysiology and the expression of hypoxia-regulated proteins are underexplored for this cancer type. Imaging with magnetic resonance imaging (MRI) and positron emission tomography (PET) has been used to probe pathophysiological aspects of the breast cancer microenvironment in vivo, including perfusion and hypoxia [13–15]. Dynamic contrast–enhanced (DCE) MRI has been widely employed in clinical studies for the characterisation of tumour vascular function [16], whilst PET with [^18^F]-labelled nitroimidazoles, such as [^18^F]-fluoromisonidazole ([^18^F]-FMISO), can provide measures of intracellular hypoxia [17, 18]. [^18^F]-FMISO-PET has shown prognostic potential in patients with ER + tumours [19] as well as utility in predicting response to primary endocrine [20] and anti-angiogenic treatment [21, 22]. Yet, despite the prognostic relevance of hypoxia in ER + disease, there is a paucity of clinical imaging studies in ER + breast tumours relating functional parameters of the tumour microenvironment to histopathological evidence or other biomarkers, with only three reports comparing [^18^F]-FMISO-PET with HIF-1α immunohistochemistry [20] or tumour-secreted cytokine expression [19, 22].

The aim of this study was to complement information from the immunohistochemical expression of endogenous markers of hypoxia (HIF-1α, CAIX) and vascularity (CD31) with imaging parameters of hypoxia and vascular function from simultaneous [^18^F]-FMISO-PET/MRI in treatment-naïve ER + breast cancer. Given that functional imaging and immunohistochemistry probe different aspects of the tumour hypoxic microenvironment—[^18^F]-FMISO-PET and DCE-MRI interrogate intracellular hypoxia and perfusion, respectively, whilst immunohistochemistry reports on hypoxia-mediated molecular events [18]—exploring associations between parameters determined from these techniques may provide additional insight into hypoxia in this cancer type for disease characterisation. To our knowledge, this study is the first to correlate in vivo imaging parameters from [^18^F]-FMISO-PET and DCE-MRI with immunohistochemical markers of hypoxia and vascularity in ER + breast cancer.

## Methods

### Study participants

Women aged  > 18 years with biopsy-confirmed primary breast cancer  > 10 mm in diameter on mammography and/or ultrasound and undergoing surgery as the first line of treatment were included in this prospective study (February 2017–November 2018). Exclusion criteria included previous history of surgery or radiotherapy for cancer, benign breast disease, inadequate renal function, pregnancy, lactation, and contraindications to MRI. The study was approved by a National Research Ethics Committee (NRES Committee East of England – Cambridge Central, 14/EE/0145) and the Administration of Radioactive Substances Advisory Committee (ARSAC), UK. Written, informed consent was provided by all study participants.

### PET/MRI acquisition

PET/MR examinations were performed on a SIGNA PET/MR scanner (GE Healthcare) as previously described [15]. In brief, participants underwent a 60-min simultaneous PET/MR scan of the breasts 120 min post injection (p.i.) of a target activity of 300 MBq [^18^F]-FMISO. Emission data were reconstructed in 12 × 5-min image frames using time-of-flight ordered-subsets expectation–maximisation with data corrections as implemented on the scanner (Supplemental Methods). Plasma radioactivity concentration from two venous blood samples obtained at the start and end of the PET/MR scan was used to scale an [^18^F]-FMISO population-based arterial input function [15], allowing calculation of the [^18^F]-FMISO influx rate constant, *K*_i_, by Patlak plot analysis [23]. The MRI protocol included the manufacturer’s two-point Dixon sequence for PET attenuation correction, T_1_- and T_2_-weighted images, and a DCE series (Supplemental Table 1). DCE-MRI involved acquisition of five pre-contrast image volumes, followed by 43 phases after intravenous bolus injection of 0.1 mmol/kg of Gadovist (Bayer Healthcare). B_1_^+^ transmission field non-uniformity was measured using a Bloch-Siegert sequence, whilst the variable flip angle (VFA) method was used for measurement of baseline T_1_, as required for the pharmacokinetic analysis of DCE-MRI data (Supplemental Methods) [15, 24].

### Image analysis

Three radiologists (1, 3, and  > 20 years of experience in breast MRI, respectively) reviewed the MRI examinations and identified lesions in consensus. Tumour regions were manually delineated in OsiriX, version 8.0.2 (Pixmeo SARL) on the peak-enhancing volume of the DCE-MRI series on all axial sections encompassing the enhancing tumour mass and including multifocal/multicentric disease. Bilateral cancers were regarded as independent lesions [25].

#### DCE-MRI

Pharmacokinetic analysis of DCE-MRI series was performed in MIStar, v3.2.63 (Apollo Medical Imaging) using the extended Tofts model to calculate the following: contrast influx rate constant, *K*^trans^; efflux rate constant, *k*_ep_; extravascular-extracellular volume fraction, *v*_e_; and plasma volume fraction, *v*_p_. Additionally, the enhancing tumour volume (ETV) was calculated using the signal enhancement ratio method with thresholds of  > 70% and  > 100% for early percent enhancement and signal enhancement ratio, respectively (Supplemental Methods) [26].

#### PET

PET images from 150 to 180 min p.i. were visually evaluated by a nuclear medicine physician and a radiologist (> 20 and 1 year of experience in PET imaging, respectively) in consensus. [^18^F]-FMISO uptake in lesions was visually compared to that in surrounding breast tissue and graded using a 4-point scale (0 = uptake lower than/equal to fibroglandular tissue; 1 = mildly increased; 2 = moderately increased; 3 = high/marked uptake). Following registration to the peak-enhancing volume of the DCE-MRI series, image frames from the entire acquisition duration (i.e. 120–180 min p.i.) were used for the determination of the [^18^F]-FMISO influx rate constant, *K*_i_, as a more specific measure of tumour hypoxia by Patlak analysis (Supplemental Methods) [23, 27]. Registered frames from 150 to 180 min p.i. were averaged and used for the determination of [^18^F]-FMISO mean and maximum standardised uptake values normalised by body weight (SUV_mean_, SUV_max_), and maximum tumour-to-muscle (*T*_max_/*M*) ratio within the tumour regions defined on the DCE-MRI. The mean radioactivity concentration in bilateral regions of the pectoral muscle was used to define normoxic tissue for *T*_max_/*M* calculations.

### Histopathology and immunohistochemistry

Histopathological information including tumour pathological size, histological subtype, grade, oestrogen receptor (ER), progesterone receptor (PR), and human epidermal growth factor receptor-2 (HER2) status were obtained from surgical pathology reports. Immunohistochemistry was performed on representative 3-µm-thick formalin-fixed and paraffin-embedded (FFPE) tumour sections, which were stained for CD31, HIF-1α, and CAIX on a BOND III autostainer (Leica Biosystems) using previously optimised conditions (Supplemental Table 2). All stained tumour sections were visually evaluated by two breast pathologists blinded to the imaging variables. For HIF-1α and CAIX, staining intensity was scored from 0 to 3 (0 = absent, 1 = mild, 2 = moderate, 3 = strong), and multiplied by the percentage of positive cells stained to generate a histoscore. CD31-stained slides were digitised on an Aperio AT2 scanner (Leica Biosystems) at × 40 magnification with a resolution of 0.25 µm/pixel, and the following parameters were obtained using the HALO image analysis software (Indica Labs): total number of microvessels, median vessel diameter (μm), and microvessel density (MVD; number of vessels/mm^2^). The overall pattern of staining distributions was classified as either diffuse or heterogeneous when staining was accentuated focally in central or peripheral areas of the section.

### Statistical analysis

Statistical analysis was performed using jamovi, version 1.2.26 (The jamovi project, 2020) or RStudio, version 1.3.1370 (RStudio Team, 2020). Continuous data were assessed for normality using the Shapiro-Wilk test. Correlations between ordinal, or ordinal and continuous variables were assessed using Kendall’s *τ*_b,_ and Spearman’s *ρ* when continuous variables were used. Associations between imaging and clinicopathological variables were examined using linear regression or mixed-effects models with a hierarchical data structure and random intercepts for subjects. Negative binomial or zero-inflated negative binomial regression was used where the response variable consisted of count (CD31 microvessel count) or discrete continuous (HIF-1α or CAIX histoscores) data as appropriate. Where the ensuing residuals from linear regression or mixed models were not normally distributed as indicated by normality tests, dependent variables (i.e. continuous PET/MR variables) were logarithmically transformed to yield more normally distributed residuals. Regression results were reported as slope coefficients ± standard error (SE) or absolute percent change with 95% confidence intervals (CI) for log-transformed variables. Effect sizes are given as incidence rate ratios (IRR) with 95% CI for negative binomial and zero-inflated models, or *R*^2^ for linear regression. *p* values  < 0.05 were considered statistically significant.

## Results

The study population comprised 22 women with 25 biopsy-confirmed, ER + breast cancers. PET/MRI data from two participants (two cancers) were excluded from correlations with immunohistochemistry due to inadequate acquisition of DCE-MRI and poor pharmacokinetic model-fitting, respectively. Sufficient diagnostic tissue material for immunohistochemistry was available for 22 tumour samples (20 participants). CD31 and CAIX expression data were available for all 22 tumour samples, whereas HIF-1α immunohistochemistry was performed in 21/22 (95%) cancers.

Tumour characteristics are summarised in Table 1. All cancers were ER + with the majority (14/22; 64%) being invasive ductal carcinomas (IDC), grade 2 or 3 (19/22; 86%), and negative for HER2 (17/22; 77%).

**Table 1:** Characteristics of tumours (*n* = 22) with an immunohistochemistry outcome

Characteristics	*n* (%)
Age at diagnosis (years)^a,b^	60 ± 12
Histological subtype	
Invasive ductal carcinoma (IDC)	14 (64)
Invasive lobular carcinoma (ILC)	4 (18)
Mixed^c^	2 (9)
Invasive mucinous carcinoma (IMC)	2 (9)
Nuclear grade	
1	3 (14)
2	10 (45)
3	9 (41)
Molecular subtype	
ER + /HER2 −	17 (77)
ER + /HER2 +	5 (23)
Carcinoma in situ	
Absence	4 (18)
Presence	18 (82)
Necrosis	
Absence	19 (86)
Presence	3 (14)
Nodal (N) status^a^	
Negative	12 (60)
Positive	8 (40)
Pathological tumour size (mm)^d^	22 [10–63]
Tumour longest diameter on MRI (mm)^d,e^	25.6 [10–60]
Tumour volume (cm^3^)^d,f^	2.19 [0.29–21.17]
Enhancing tumour volume (cm^3^)^d,f^	1.85 [0.29–12.95]

^a^Calculated in *n* = 20 patients

^b^Data presented as mean ± standard deviation (SD)

^c^Cancers with the presence of both ductal and lobular components

^d^Data presented as median [range]

^e^Pathological tumour size was defined as the largest tumour diameter measured on surgical specimens

^f^Calculated in *n* = 20 cancers, for which both DCE-MRI and immunohistochemistry data were available

*ER* + , oestrogen receptor–positive; *HER2 − *, human epidermal growth factor 2-negative; *HER2* + , human epidermal growth factor 2-positive

### Expression of CD31, HIF-1α, and CAIX and relationships with clinicopathological variables

Of the 22 cancers stained with CD31, 50% (11/22) showed heterogeneous expression patterns, with 6/11 (54%) of these exhibiting pronounced staining in the periphery of the lesion (Table 2). The number and diameter of tumour microvessels associated positively with pathological size (IRR [95% CI]: 1.02 [1.01–1.04], *p* = 0.002) and HER2 negativity (*R*^2^ = 0.27, *p* = 0.01), respectively; no significant correlations were observed with other clinicopathological variables (Supplemental Figs. 1–3).

**Table 2:** Summary of immunohistochemistry results for CD31, HIF-1α, and CAIX

Characteristic	Metric
CD31 [*n* = 22 lesions]	
Microvessel count (number)^a^	15,643 [2364–49,074]
Microvessel density (MVD; vessels/mm^2^)^b^	50.3 ± 21.9
Microvessel diameter (μm)^b^	10.10 ± 0.75
Staining distribution	
Diffuse, *n* (%)	11 (50)
Heterogeneous, *n* (%)	11 (50)
HIF-1α [*n* = 21 lesions]	
Intensity score	
0 – No staining, *n* (%)	4 (19)
1 – Mild, *n* (%)	13 (62)
2 – Moderate, *n* (%)	4 (19)
3 – Strong, *n* (%)	0 (0)
% Staining^a^	5 [0–20]
Histoscore^a^	0 [0–40]
Staining distribution [*n* = 17 positive lesions]	
Diffuse, *n* (%)	6 (35)
Heterogeneous, *n* (%)	11 (65)
CAIX [*n* = 22 lesions]	
Intensity score	
0 – No staining, *n* (%)	16 (73)
1 – Mild, *n* (%)	0 (0)
2 – Moderate, *n* (%)	2 (9)
3 – Strong, *n* (%)	4 (18)
% Staining^a^	0 [0–20]
Histoscore^a^	5 [0–40]
Staining distribution [*n* = 6 positive lesions]	
Diffuse, *n* (%)	2 (33)
Heterogeneous, *n* (%)	4 (67)

^a^Data presented as median [range]

^b^Data presented as mean ± SD

HIF-1α staining was found in 17/21 (80%) lesions, which was characterised as either mild or moderate (Table 2). Heterogeneous staining distributions were observed in 11/17 (65%) tumours, with 8/11 (72%) lesions displaying accentuated staining in the central portion of the specimen (Table 2). Cancers with an in situ component had higher HIF-1α histoscores (IRR [95% CI]: 4.84 [0.93–20.2], *p* = 0.04); associations with other clinical parameters were not significant (Supplemental Fig. 4).

CAIX was detectable in 6/22 (27%) tumours (Table 2). Of these, 4/6 (66%) cancers displayed heterogeneous expression patterns with pronounced staining in central areas of the specimen (Table 2). CAIX expression associated with larger tumour size (IRR [95% CI]: 1.02 [1.01–1.03], *p* < 0.001) and HER2 positivity (IRR [95% CI]: 1.65 [1.15–2.35], *p* = 0.006) (Supplemental Fig. 5). Additionally, the percentage of positive cells stained associated positively with tumour grade (IRR [95% CI]: 1.51 [1.10–2.91], *p* = 0.01).

**Fig. 1:**
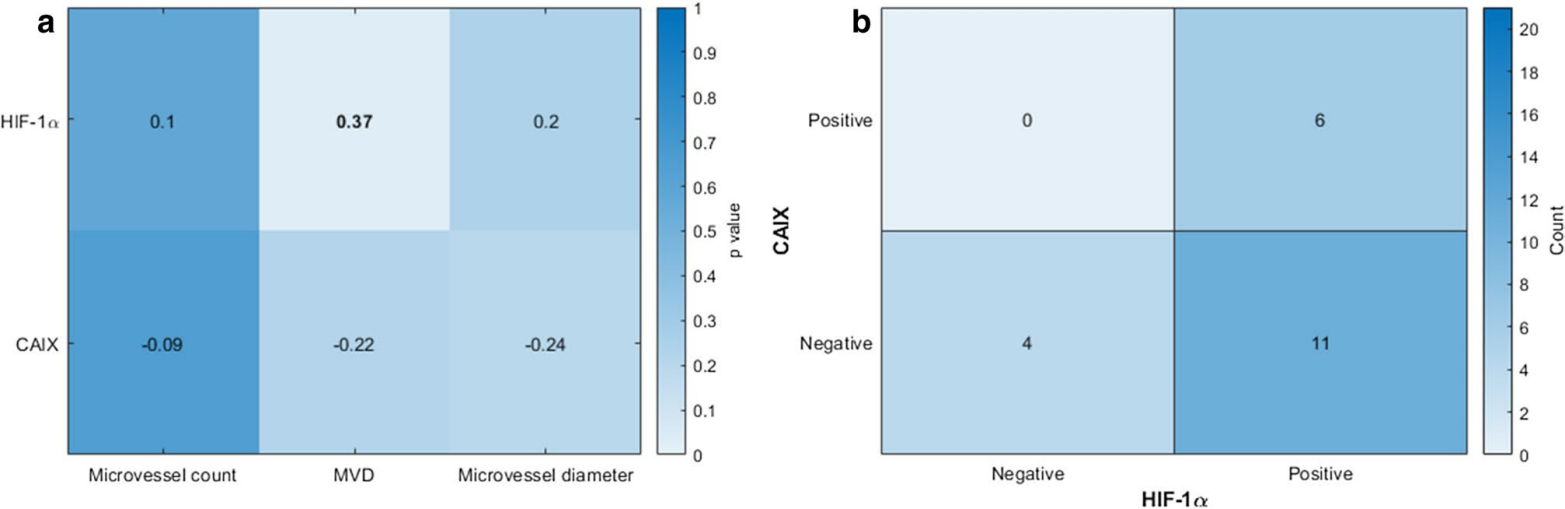
Associations between HIF-1α and CAIX expression. **a** Correlations (Kendall’s *τ*_b_) between HIF-1α or CAIX histoscores and CD31 parameters. **b** Cross-tabulation of HIF-1α and CAIX immunohistochemistry results

HIF-1α expression associated positively with MVD, whilst negative correlations were found between CAIX expression and CD31 vascularity indices, which were not statistically significant (Fig. 1a). No significant correlation was observed between HIF-1α and CAIX histoscores (*ρ* = 0.20, *p* = 0.39), with 6/17 (35%) HIF-1α-positive tumours showing positive CAIX expression (Fig. 1b).

### Relationships between immunohistochemistry markers and ^18^F-FMISO-PET/MR imaging indices

Figure 2 illustrates representative examples of two cancers stained with HIF-1α, CAIX, and CD31 together with parametric images indicating hypoxia (*K*_i_) and perfusion (*K*^trans^) from [^18^F]-FMISO-PET and DCE-MRI, respectively. Representative static [^18^F]-FMISO-PET images (150–180 min p.i.) alongside the corresponding DCE-MRI phase at peak enhancement are shown in Fig. 3. Results from the visual analysis of [^18^F]-FMISO uptake in tumours are given in Table 3.

**Fig. 2:**
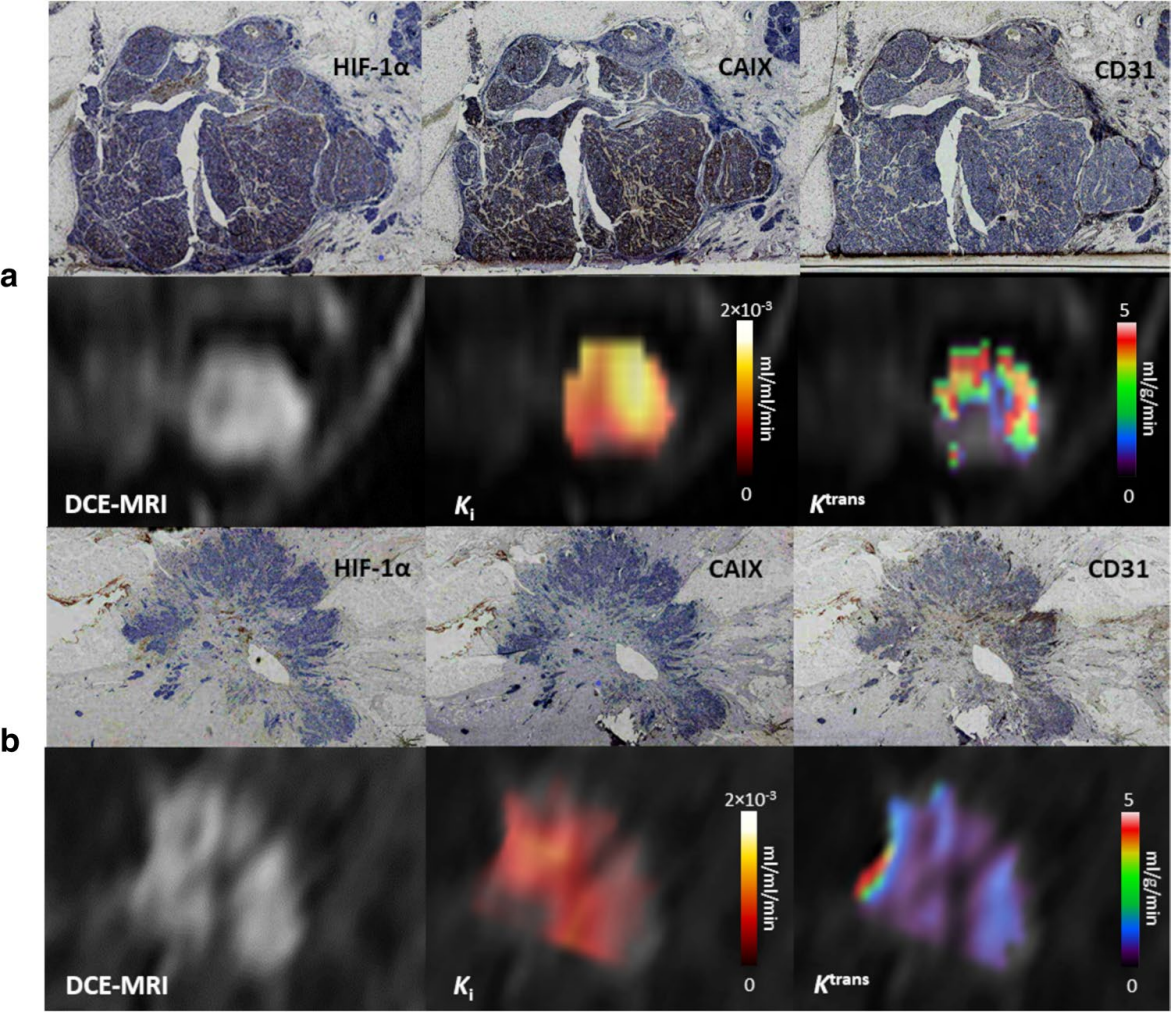
Representative images of HIF-1α, CAIX, and CD31 staining in tumour sections of two representative ER + breast cancers together with parametric maps indicating hypoxia (*K*_i_) and perfusion (*K*^trans^) from [^18^F]-FMISO-PET and DCE-MRI, respectively. Extra-tumoural areas on *K*_i_ and *K*^trans^ maps have been masked to only indicate values inside the tumour. Immunostaining for HIF-1α, CAIX and CD31 can be seen in brown colour. **a** Invasive mucinous carcinoma (IMC) with ductal carcinoma in situ (DCIS), grade 2, ER + /HER2 − with a diffuse staining pattern of mild intensity for HIF-1α (histoscore: 5) and strong CAIX immunostaining throughout the tumour section (histoscore: 40). Moderate vascularity can be interpreted from CD31 (MVD: 40.6 vessels/mm^2^), which was mainly observed in peripheral areas of the section. The [^18^F]-FMISO *K*_i_ map revealed the presence of hypoxia in the entire tumour region. Areas of increased perfusion were observed towards the edges of the tumour on the *K*^trans^ map. **b** Invasive ductal carcinoma (IDC) with DCIS, grade 3, ER + /HER2 + with mild heterogenous staining for HIF-1α (histoscore: 2) and negative CAIX expression. CD31 staining indicated moderate vascularity (MVD: 40.0 vessels/mm^2^), which was prominent in areas of increased HIF-1α expression. Mild-moderate hypoxia was observed on the ^18^F-FMISO *K*_i_ map corresponding to regions of hypoperfusion on the *K*^trans^ map

**Fig. 3:**
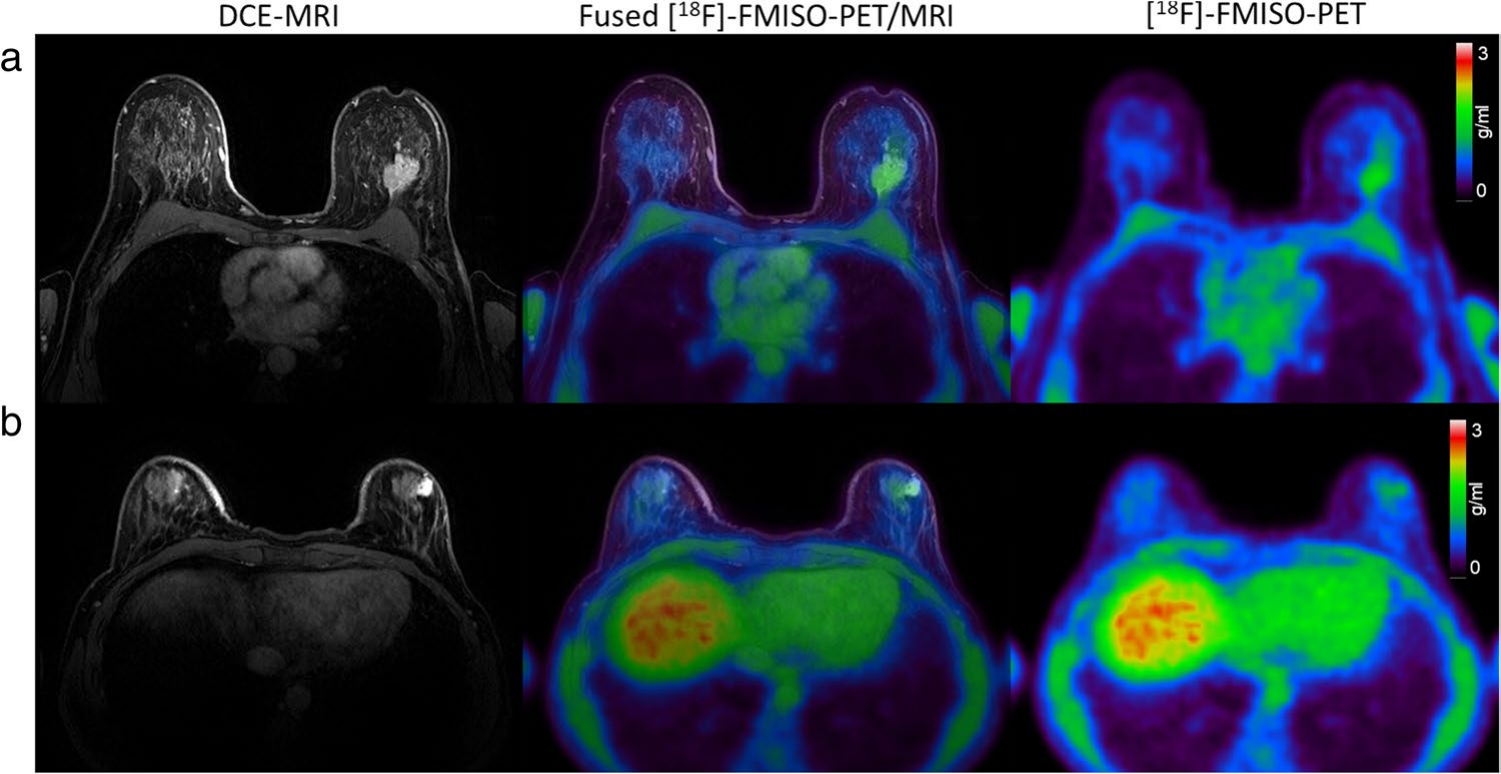
Representative transaxial images from two patients with ER + /HER2 − breast cancer. *Left*: Dynamic contrast-enhanced (DCE) MRI at peak enhancement; *centre*: [^18^F]-FMISO-PET (150–180 min p.i.) overlaid on the peak-enhancing DCE-MRI; *right*: [^18^F]-FMISO-PET (150–180 min p.i.). **a** Invasive ductal carcinoma (IDC), grade 2 with high [^18^F]-FMISO uptake in the tumour (SUV_max_ = 1.9; SUV_mean_ = 1.4; *T*_max_/*M* = 1.5). **b** Invasive mucinous carcinoma (IMC) with ductal carcinoma in situ (DCIS), grade 3, with moderate [^18^F]-FMISO uptake in the tumour (SUV_max_ = 1.2; SUV_mean_ = 1.0; *T*_max_/*M* = 1.0)

**Table 3:** Summary of results from the visual analysis of [^18^F]-FMISO uptake in tumours with an immunohistochemistry outcome (*n* = 22 lesions)

Characteristic	Metric
^18^F-FMISO visual score [*n* = 22 lesions]	
0 – Lower than/equal to surrounding tissue	2 (9%)
1 – Mildly increased	3 (14%)
2 – Moderately increased	12 (54%)
3 – High/marked	5 (23%)

**Fig. 4:**
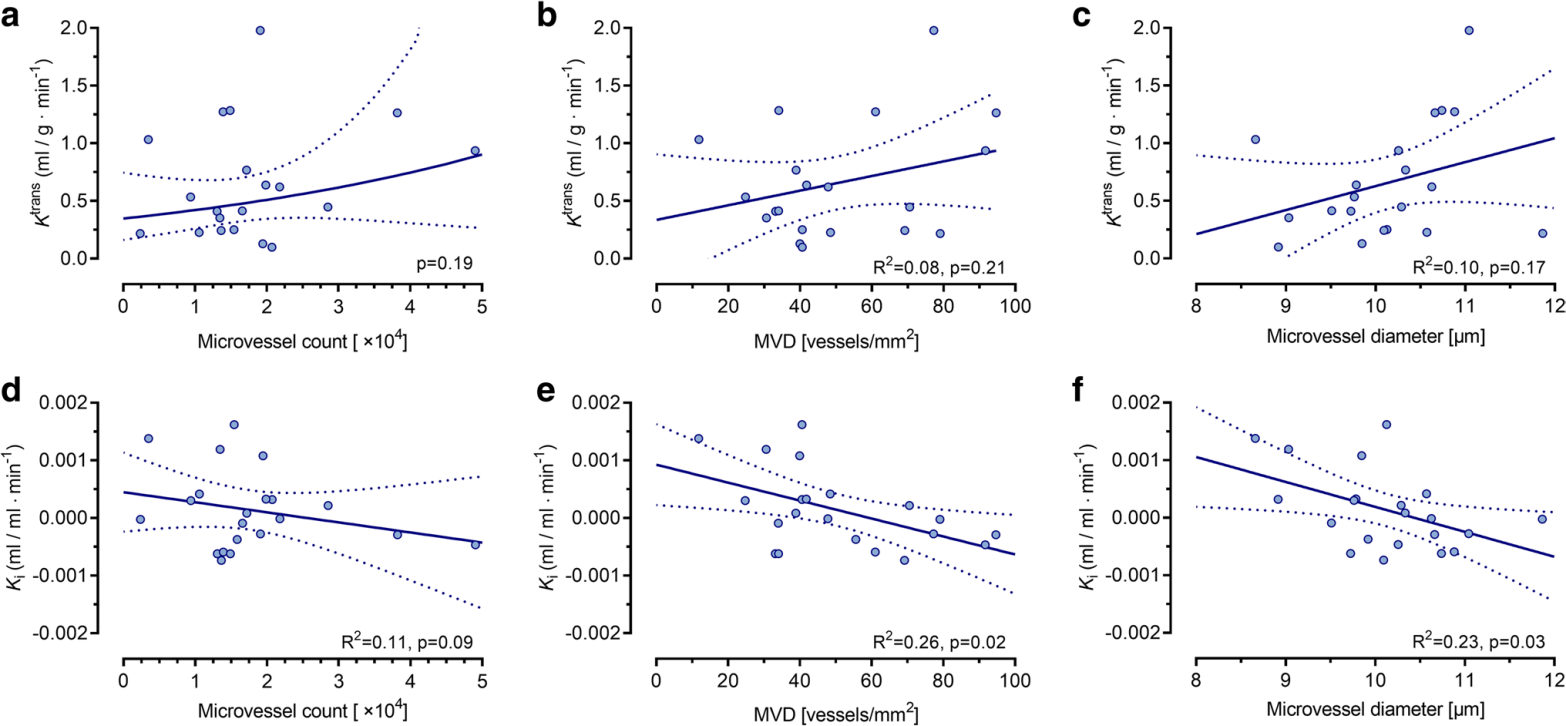
Scatter plots and regression lines for associations between PET/MR imaging variables and CD31 microvascular parameters. **a**–**c** Contrast influx rate constant *K*^trans^ (mL/g/min) and (**d**–**f**) [^18^F]-FMISO influx rate constant *K*_i_ (mL/mL/min) *vs* microvessel count, microvessel density (MVD; vessels/mm^2^), and microvessel diameter (μm), respectively

Except for a significant positive association between the number of tumour microvessels and ETV (slope ± SE: 0.01 ± 0.18, *p* = 0.02), pharmacokinetic parameters from DCE-MRI did not significantly correlate with CD31 measures (Fig. 4a–c; Supplemental Table 3). In contrast, negative relationships were observed between [^18^F]-FMISO *K*_i_ and CD31 vascular parameters (Fig. 4d–f), which were statistically significant for MVD (slope ± SE: − 0.016 ± 0.006, *R*^2^ = 0.26, *p* = 0.02) and vessel diameter (slope ± SE: − 0.43 ± 0.18, *R*^2^ = 0.23, *p* = 0.03) (Supplemental Table 3).

**Fig. 5:**
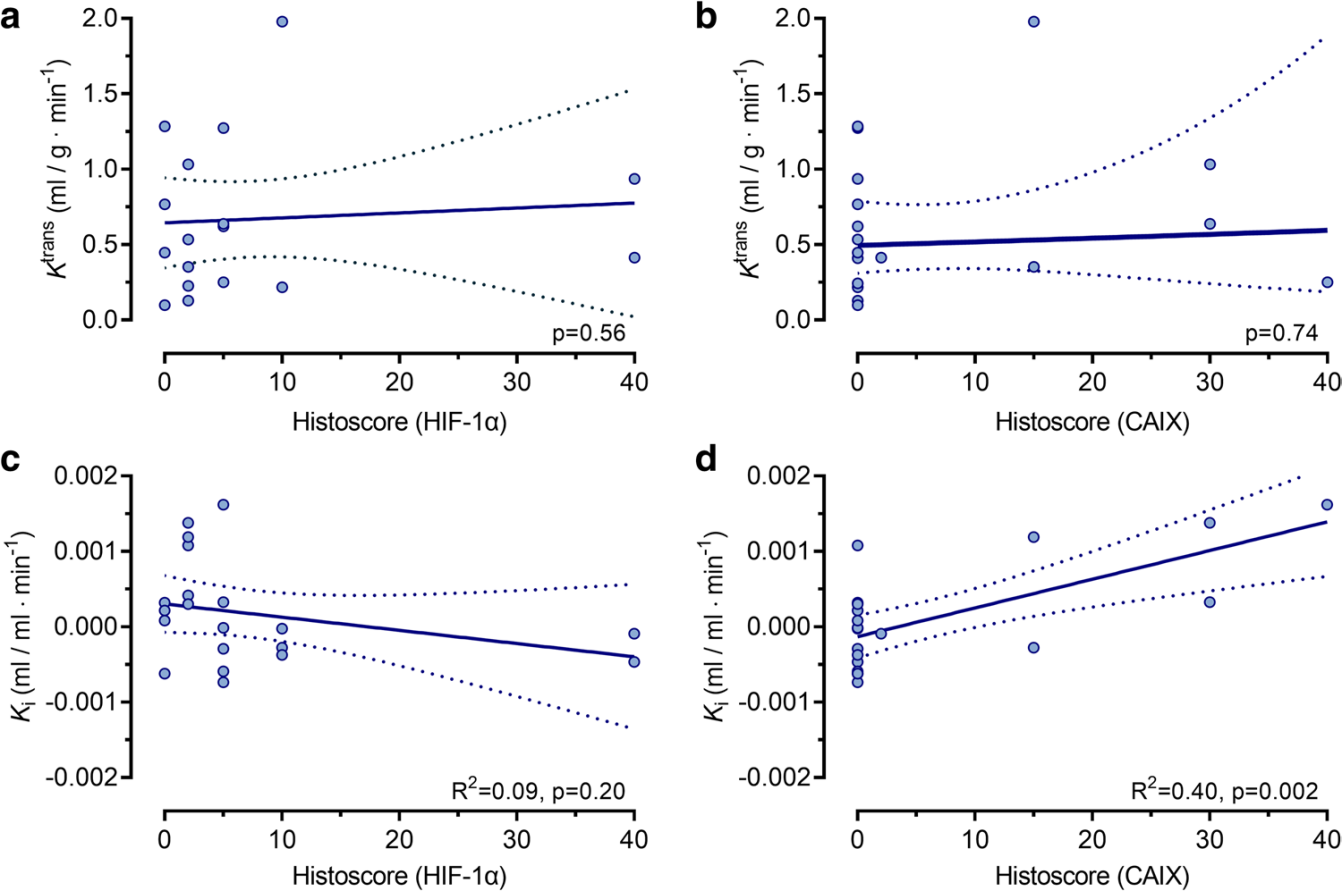
Scatter plots and regression lines for associations between PET/MR imaging variables vs HIF-1α and CAIX expression. **a**, **b** Contrast influx rate constant *K*^trans^ (mL/g/min) and (**c**, **d**) [^18^F]-FMISO influx rate constant *K*_i_ (mL/mL/min) vs HIF-1α or CAIX histoscore, respectively

DCE-MRI parameters showed no significant relationships with HIF-1α or CAIX histoscores (Fig. 5a, b; Supplemental Table 4), aside from ETV which was positively associated with HIF-1α expression (slope ± SE: 0.11 ± 0.04, *R*^2^ = 0.24, *p* = 0.01), whilst a negative relationship was observed with CAIX (slope ± SE: − 0.13 ± 0.04, *R*^2^ = 0.17, *p* = 0.01). [^18^F]-FMISO *K*_i_ showed a positive association with CAIX histoscore (slope ± SE: 1.3 × 10^−4^ ± 7.9 × 10^−3^, *R*^2^ = 0.40, *p* = 0.002), whilst no significant associations were observed between PET parameters and HIF-1α expression (Fig. 5c, d; Supplemental Table 4). Additionally, [^18^F]-FMISO visual scores were not significantly correlated with immunohistochemistry results (Supplemental Table 5).

## Discussion

A key aim of this study was to explore associations between imaging parameters from [^18^F]-FMISO-PET/MRI and endogenous immunohistochemical markers of hypoxia and vascularity in ER + breast cancer. Hypoxia, measured by the [^18^F]-FMISO influx rate constant *K*_i_, was associated with increased CAIX expression, low MVD, and smaller vessel diameters. Additionally, we found a negative relationship between enhancing tumour volume (ETV) from DCE-MRI and CAIX expression. These findings are consistent with the notion that morphologic and functional abnormalities of the tumour microvasculature are the major determinants of hypoxia in cancers [28] and corroborate the previously reported perfusion-driven character of hypoxia in breast carcinomas [15].

In agreement with previous reports, CAIX expression was positively correlated with tumour size, grade, and HER2 positivity [11, 29], whilst the presence of in situ carcinoma associated with HIF-1α positivity [30]. Staining distributions for HIF-1α and CAIX were predominantly heterogeneous and mainly confined to the central portion of tumours, as previously described for breast cancer [31]. Irregular spatial relationships were found between CD31 staining patterns and those of CAIX or HIF-1α, with several samples showing colocalisation of CD31 and hypoxia-related proteins. The presence of HIF-1α in regions with high vascular density is consistent with the HIF-dependent upregulation of angiogenic factors [1]. Additional pathophysiological mechanisms, including the metabolic state of the tumour, long oxygen diffusion distances, and interstitial fluid pressure, may explain the co-existence of hypoxia in highly vascularised regions [32, 33].

Whilst increased CAIX expression correlated with [^18^F]-FMISO *K*_i_, no associations were observed between HIF-1α and PET hypoxia indices. Furthermore, we observed a weak correlation between HIF-1α and CAIX expression, with the majority of HIF-1α-positive lesions lacking co-expression of CAIX. Although changes in tumour oxygen levels between imaging and surgery may have contributed to the poor association between PET variables and HIF-1α expression, our findings are in keeping with previous studies in various tumours, including breast cancer, reporting the absence of a significant association between HIF-1α and hypoxia [34, 35] or HIF-1α-related proteins [35–37]. Notably, in several types of cancer, including ER + breast malignancy, various oncogenic signalling mechanisms have been shown to activate HIF-1α-related pathways independent of hypoxic stimulation [6, 37–40]. Since CAIX overexpression is the generally accepted sequela of hypoxia-induced HIF-1α activation [41], our results strengthen the notion that the overexpression of HIF-1α in primary ER + breast cancer may be largely hypoxia-independent. This assertion is further corroborated by the observation that most of our cancer samples were HIF-1α-positive despite the absence of necrosis [42]. In this context, several authors have postulated that CAIX may provide a more reliable marker of hypoxia in tumours than HIF-1α, given its strong regulation by hypoxia-related processes and long half-life after hypoxic induction, allowing for the identification of chronically hypoxic tumour areas [43].

Consistent with the proangiogenic role of HIF-1α, we observed a positive association between HIF-1α expression and both MVD and ETV. Furthermore, there was a tendency for a negative association between CAIX and CD31 immunohistochemistry. Although pharmacokinetic parameters from DCE-MRI exhibited positive and negative associations with HIF-1α and CAIX expression, respectively, our results did not demonstrate statistically significant relationships. Given that ER + breast tumours are generally characterised by lower blood flow [44] and potentially perfusion-driven hypoxia [15], the lack of a significant association between DCE-MRI functional metrics and hypoxia-related proteins suggests that the oxygenation status of this breast tumour type may be largely but not uniquely defined by tumour perfusion. Our findings are supported by previous research in prostate [45, 46], head-and-neck [47], and endometrial cancers [48], and further demonstrate that hypoxia is a combination of tumour-intrinsic and microenvironment-related factors [47].

Similarly, no significant relationships were observed between DCE-MRI parameters and CD31 immunohistochemistry, although ETV positively associated with the number of tumour microvessels. Earlier studies exploring associations between DCE-MRI and immunohistochemical markers of vascularity in breast tumours have demonstrated conflicting results, with several authors reporting significant correlations with semi-quantitative metrics [49–52], whilst variable associations were observed with pharmacokinetic parameters [53–57]. The inconsistent relationships between histologic measures of vascularity and pharmacokinetic parameters from DCE-MRI between various studies can be partially explained by differences in metrics used for DCE-MRI quantification, and the inability to accurately register 2D images of histopathological samples with corresponding slices from 3D imaging data, as well as the absence of a standardised method for vascularity quantification on immunohistochemistry, with antibody type and measurement methods being variable amongst studies. However, it should also be noted that histologic measures of vascularity are not precisely related to the functional properties of the tumour microvasculature, which are critical for the interpretation of DCE-MRI pharmacokinetic results [16, 57].

The main limitation of our study was the small sample size, predominantly comprising ductal cancers. Although our findings should be confirmed in a larger breast cancer cohort, it should be noted that ER + IDC represents the most common histological subtype of breast carcinoma with a higher tendency for hypoxia and the expression of hypoxia-related proteins [29, 30]. Furthermore, comparison of whole-tumour imaging metrics vs single-slice histology parameters did not permit the assessment of intratumoural heterogeneity.

In conclusion, in ER + breast cancer, hypoxia measured by [^18^F]-FMISO-PET associated negatively with MVD and microvessel diameter derived from CD31 immunohistochemistry, whilst a positive correlation was observed with CAIX expression. No relationships were observed between DCE-MRI pharmacokinetic metrics and immunohistochemical markers. The combination of multimodal in vivo imaging and immunohistochemistry facilitates interrogation of different aspects of the tumour pathophysiology, with multimodal imaging also providing assessment of the whole tumour. Taken together, the data presented here can be viewed as providing indication of the benefit of non-invasive multimodal assessment of the tumour microenvironment, which may complement information from histopathology in providing additional disease characterisation or evaluating therapeutic response.

## Supplementary Information

Below is the link to the electronic supplementary material.

Supplementary file1 (PDF 708 KB)

## Abbreviations

[^18^F]-FMISO: [^18^F]-fluoromisonidazole
CAIX: Carbonic anhydrase IX
CD31: Cluster of differentiation 31
ER: Oestrogen receptor
HIF-1α: Hypoxia-inducible factor 1α
*k*_ep_: Contrast efflux rate constant (min^−1^)
*K*_i_: Tracer influx rate constant (mL/mL/min)
*K*^trans^: Contrast influx transfer rate constant (mL/g/min)
MVD: Microvessel density (vessels/mm^2^)
SUV: Standardised uptake value (g/mL)
*T*_max_/M: Maximum tumour-to-muscle ratio
*v*_e_: Extravascular-extracellular volume fraction
*v*_p_: Plasma volume fraction

## Declarations

### Guarantor

The scientific guarantor of this publication is Professor Fiona J. Gilbert.

### Statistics and biometry

No complex statistical methods were necessary for this paper.

### Study subjects or cohorts overlap

Some study subjects have been previously reported in Carmona-Bozo JC, Manavaki R, Woitek R, et al (2021) Hypoxia and perfusion in breast cancer: simultaneous assessment using PET/MR imaging. Eur Radiol 31:333-44.

### Methodology

• prospective

• observational

• performed at one institution
