# Supplementary material for: PET/MRI of hypoxia and vascular function in ER-positive breast cancer: correlations with immunohistochemistry
Source: Eur Radiol. 2023 May 11;33(9):6168–78. doi: 10.1007/s00330-023-09572-6 (PMC10415421; doi:10.1007/s00330-023-09572-6)
Supplement: Supplementary file 1 — Supplementary file1 (PDF 708 KB) [file 330_2023_9572_MOESM1_ESM.pdf]

Supplementary Material

I. Methods

A. PET/MR acquisition

Participants underwent a PET/MR scan of the breasts in the prone position, using a 16-channel bilateral breast coil (RAPID Biomedical), 120.3 [119.8 – 127.5] min after injection of 315 ± 12 MBq [^{18}F]-FMISO. The uptake period post injection (p.i.) was used to enhance hypoxic tissue contrast and additionally allow for the free [^{18}F]-FMISO concentrations in tissue and blood to reach equilibrium [25,26]; the latter being a requirement for the determination of influx rate constant K_i using Patlak analysis.

Emission data were reconstructed into a 192×192×89 matrix with 3.12×3.12×2.78-mm voxels using time-of-flight ordered-subsets expectation maximisation (TOF-OSEM). Image reconstruction utilised 4 iterations and 28 subsets, together with an isotropic 4-mm full-width-at-half-maximum (FWHM) Gaussian filter post reconstruction. Corrections for normalisation, dead-time, random events, scatter, attenuation, sensitivity, and isotope decay were applied as implemented on the scanner.

MRI sequence information is given in Supplemental Table 1.

B. Image analysis

DCE-MRI: Prior to pharmacokinetic analysis, a rectangular volume encompassing the entire tumour across the DCE-MRI series was motion-corrected in MIStar (Apollo Medical Imaging) using affine registration. B_1^+-correction maps were produced from the Bloch-Siegert method using in-house software implemented in Matlab R2016b (Mathworks Inc). T_{10} maps were calculated in MIStar, using the B_1^+-correction maps to correct for spatial variations in flip angle. Both the B_1^+-correction maps and corrected T_{10} maps were subsequently resampled to DCE-MRI space (i.e., 512×512×112; Supplemental Table 1) for use in pharmacokinetic analysis [24]. Pharmacokinetic modelling was performed in MIStar using the modified Fritz-Hansen arterial input function with all model parameters restricted to positive values and an upper limit for v_e of 1. Where modelling resulted in K^{trans} values outside the physiological range (0.001 – 5 ml/g/min) [29], voxels were excluded from regional analyses.

In addition to pharmacokinetic analysis, the enhancing tumour volume (ETV) was calculated for each patient using the signal enhancement ratio method [28]. Early percent enhancement (PE_e) and signal-enhancement ratio (SER) maps for each lesion were derived from pre-contrast images (S_0), early post-contrast (S_E, phase 19 of the DCE-MRI series) and late post-contrast (S_L, phase 46 of the DCE-MRI series) images, using the following equations: $PE_e = 100\times(S_E - S_0)/S_0$ and $SER = 100\times(S_E - S_0)/(S_L - S_0)$, respectively. ETV was subsequently calculated by summing all image voxels within the tumour ROI above the thresholds for PE_e and SER, followed by connectivity test to eliminate single voxels or very small regions. For ETV calculations, thresholds of 70% and 100% were used for PE_e and SER respectively [28].

PET: As increased [^{18}F]-FMISO uptake (i.e., SUV, T_{max}/M ratio) may represent elevated tracer delivery to a tumour rather than trapping under hypoxic conditions, the influx rate of [^{18}F]-FMISO (K_i) into the trapped (hypoxic) tissue compartment was determined as a more specific measure of tumour hypoxia [30]. K_i maps were produced by Patlak analysis [16], using in-house software implemented in Matlab R2016b and an [^{18}F]-FMISO population-based arterial input function derived from existing data [15]. To reduce the effect of patient motion during acquisition on K_i estimation, [^{18}F]-FMISO dynamic image series were non-rigidly registered to the first frame using the Advanced Normalization Tools (ANTs) package (http://stnava.github.io/ANTs/). Image analysis was performed in Analyze 12.0 (AnalyzeDirect Inc.).

Supplemental Table 1: MRI acquisition parameters.

Acquisition parameters	T_1 mapping (VFA)	B_1^+ mapping (Bloch-Siegert)	DCE (VIBRANT-TRICKS)
Sequence	3D SPGR	2D SPGR	3D SPGR
Acquisition plane	Axial	Axial	Axial
FOV diameter (mm)	350	350	350
Image matrix	256×256	128×128	512×512
Slice thickness (mm)	2.8	7.0	2.8 (interpolated to 1.4)
No. of slices	112	22	112
Pixel size (mm)	1.4×1.4	2.7×2.7	0.6×0.6
Fat suppression	No	No	Yes[a]
ASSET factor	2	n/a	2.5
TR (ms)	4.2	24	7.1
TE (ms)	2.1	13.7	3.8
RF excitation (degrees)	2, 3, 5, 10, 15	20	12
No. of averages	1	1	0.5
Acquisition time	33 s (per flip angle)	2 m 20 s	8 m 5 s[b]

[a] Spatial-spectral water excitation
[b] Nominal temporal resolution: 10 s per phase
VFA: variable flip angle; DCE: dynamic contrast enhanced; VIBRANT-TRICKS: volume image breast assessment – time-resolved imaging of contrast kinetics; 3D SPGR: three-dimensional spoiled gradient recalled echo; 2D SPGR: two-dimensional spoiled gradient recalled echo; FOV: field-of-view; ASSET: array spatial sensitivity encoding technique; TR: repetition time; TE: echo time; RF: radiofrequency.

Supplemental Table 2: Characteristics of the antibodies used in immunohistochemical analyses.

**Target**	**Host | Clonality (Cat. No)**	**Type**	**Incubation time**	**Dilution | Concentration**	**Retrieval time**	**Retrieval agent**
			**min**	**μg/ml**	**min**	
**CD31**	mouse | moAB (Dako, M0823)	Primary	15	1:50 | 4.1	20	Sodium citrate
**HIF-1α**	rabbit | moAB (Abcam, ab51608)	Primary	15	1:100 | 23.36	20	Sodium citrate
**CAIX**	mouse | moAB (BioScience, AB1001)	Primary	15	1:1000 | 1	20	Sodium citrate

Cat. No.: Catalogue number

II. Results

Supplemental Table 3: Regression slope coefficients (β) with standard errors (SE) and *p*-values for associations between PET/MR imaging variables and parameters from the expression of CD31. Results from linear regression models employing logarithmic transformation of the response variable are reported as percentage change in the dependent variable with 95% confidence intervals (CI). Effect sizes (ES) from linear regression are reported as R^2.

Parameter	CD31		
	**Microvessel count** β ± SE, (ES), p	**Microvessel density (MVD)** β ± SE, (ES), p	**Microvessel diameter** β ± SE, (ES), p
DCE-MRI			
K^{trans}	0.00 [0.00 – 0.01] %, (–),0.19[a,b]	$6.3\times10^{-3} \pm 4.9\times10^{-3}$, (0.08), 0.21	0.21 ± 0.14, (0.10), 0.17
hs-K^{trans}	$4.0\times10^{-5} \pm 1.9\times10^{-5}$, (0.15), 0.05	$-2.8\times10^{-3} \pm 8.4\times10^{-4}$, (0.01), 0.73	-0.31 ± 0.24, (0.08), 0.21
k_{ep}	$8.1\times10^{-7} \pm 1.6\times10^{-6}$, (0.00), 0.63	$-1.5\times10^{-4} \pm 6.2\times10^{-4}$, (0.00), 0.81	-6.3×10^{-3} ± 0.02, (0.01), 0.74
v_e	$5.7\times10^{-6} \pm 5.3\times10^{-6}$, (0.01), 0.30	$1.6\times10^{-3} \pm 2.1\times10^{-3}$, (0.03), 0.46	0.10 ± 0.06, (0.14), 0.10
v_p	$3.4\times10^{-5} \pm 2.5\times10^{-5}$, (0.04), 0.20	1.29 [-0.77 – 3.39] %, (–), 0.21[b]	0.07 ± 0.04, (0.17), 0.07
ETV	0.01 [0.00 – 0.01], (–), 0.02*[b]	1.13 [-1.21 – 3.53] %, (–), 0.33[b]	-15.3 [-58.4 – 72.3] %, (–), 0.63[b]
PET			
K_i ($\times10^{-3}$)	$-3.0\times10^{-5} \pm 1.7\times10^{-5}$, (0.11), 0.09	$-1.6\times10^{-2} \pm 6.1\times10^{-3}$, (0.26), 0.02*	0.43 ± 0.18, (0.23), 0.03*
SUV_{mean}	$4.7\times10^{-6} \pm 6.0\times10^{-6}$, (0.00), 0.44	$-2.3\times10^{-4} \pm 2.3\times10^{-3}$, (0.00), 0.92	0.01 ± 0.10, (0.00), 0.88
SUV_{max}	$1.2\times10^{-5} \pm 7.5\times10^{-6}$, (0.00), 0.20	$-2.8\times10^{-4} \pm 3.7\times10^{-3}$, (0.00), 0.94	-0.02 ± 0.11, (0.02), 0.84
T_{max}/M	$6.6\times10^{-6} \pm 5.6\times10^{-6}$, (0.00), 0.25	$-1.2\times10^{-4} \pm 2.7\times10^{-3}$, (0.00), 0.97	-0.04 ± 0.07, (0.02), 0.58

[a] Linear regression models against CD31 microvessel count were controlled for the effect of tumour pathological size.
[b] Linear regression using log transformation of the response variable to satisfy the assumption of normality in regression residuals. Results are reported as percentage change in the dependent variable with 95% CI.
Statistical significance: p<0.05*.
K^{trans}: contrast influx rate (ml/g/min); k_{ep}: contrast efflux rate (min^{-1}); v_e: fractional volume of extravascular-extracellular space; v_p: plasma fractional volume; K_i: [^{18}F]-FMISO influx rate (ml/ml/min); SUV: standardized uptake value normalized by body weight (g/ml); T_{max}/M: maximum tumour-to-muscle ratio.

Supplemental Table 4: Regression slope coefficients (β) with standard errors (SE) and p-values for associations between PET/MR imaging variables and parameters from the expression of HIF-1α or CAIX. Results from linear regression models employing logarithmic transformation of the response variable are reported as percentage change in the dependent variable with 95% confidence intervals (CI). Effect sizes (ES) are reported as ω^2p or R^2 for multiple and linear regression, respectively.

**Parameter**	**HIF-1α**	**CAIX**
	**Histoscore** β ± SE, (ES), p	**Histoscore**[a] β ± SE, (ES), p
DCE-MRI		
K^{trans}	1.00 [-2.55 – 4.68] %, (–), 0.56[a]	0.55 [-2.84 – 4.06] %, (–),0.74[a,b]
hs-K^{trans}	0.02 ± 0.02, (0.07), 0.56	-0.01 ± 0.01, (0.06), 0.23[b]
k_{ep}	$5.8\times10^{-4} \pm 1.3\times10^{-3}$, (0.01), 0.65	$-6.9\times10^{-4} \pm 1.1\times10^{-3}$, (0.02), 0.56[b]
v_e	$-3.5\times10^{-3} \pm 4.3\times10^{-3}$, (0.04), 0.43	$-1.5\times10^{-3} \pm 4.1\times10^{-3}$, (0.01), 0.72[b]
v_p	1.17 [-3.14 – 5.66]%, (–),0.58[a]	0.86 [-3.20 – 5.08] %, (–), 0.82[a,b]
ETV	0.11 ± 0.04, (0.24), 0.01*	-0.13 ± 0.04, (0.17), 0.01*[b]
PET		
K_i ($\times10^{-3}$)	$-1.7\times10^{-2} \pm 1.3\times10^{-2}$, (0.09), 0.20	$1.3\times10^{-4} \pm 7.9\times10^{-3}$, (0.40), 0.002**[b]
SUV_{mean}	$-2.2\times10^{-3} \pm 4.6\times10^{-3}$, (0.02), 0.58	$-3.4\times10^{-3} \pm 4.2\times10^{-3}$, (0.04), 0.43[b]
SUV_{max}	$-3.5\times10^{-3} \pm 7.5\times10^{-3}$, (0.01), 0.65	$-8.6\times10^{-3} \pm 6.8\times10^{-3}$, (0.03), 0.22[b]
T_{max}/M	$-1.2\times10^{-4} \pm 5.2\times10^{-3}$, (0.00), 0.98	$-3.6\times10^{-3} \pm 5.1\times10^{-3}$, (0.02), 0.48[b]

[a] Linear regression using a log transformation of the response variable to satisfy the assumption of normality in regression residuals. Results are reported as percentage change in the dependent variable with 95% confidence intervals (CI).
[b] Linear regression models against CAIX histoscore were controlled for the effect of tumour pathological size.
Statistical significance: p<0.01**; p<0.05*.
K^{trans}: contrast influx rate (ml/g/min); k_{ep}: contrast efflux rate (min^{-1}); v_e: fractional volume of extravascular-extracellular space; v_p: plasma fractional volume; K_i: [^{18}F]-FMISO influx rate (ml/ml/min); SUV: standardized uptake value normalized by body weight (g/ml); T_{max}/M: maximum tumour-to-muscle ratio.

Supplemental Table 5: Correlation coefficients (Kendall's τ_b) and p values for relationships between [^{18}F]-FMISO visual scores and CD31, HIF-1α, and CAIX immunohistochemistry (*n*=22 lesions).

Characteristic	[^{18}F]-FMISO visual score
	τ_b, p
**CD31**	
Microvessel count	0.28, 0.08[a]
Microvessel density (MVD)	-0.03, 0.88
Microvessel diameter	0.07, 0.69
**HIF-1α**	
Histoscore	0.12, 0.53
**CAIX**	
Histoscore	0.11, 0.50[a]

[a] Partial correlation controlling for the effect of tumour pathological size

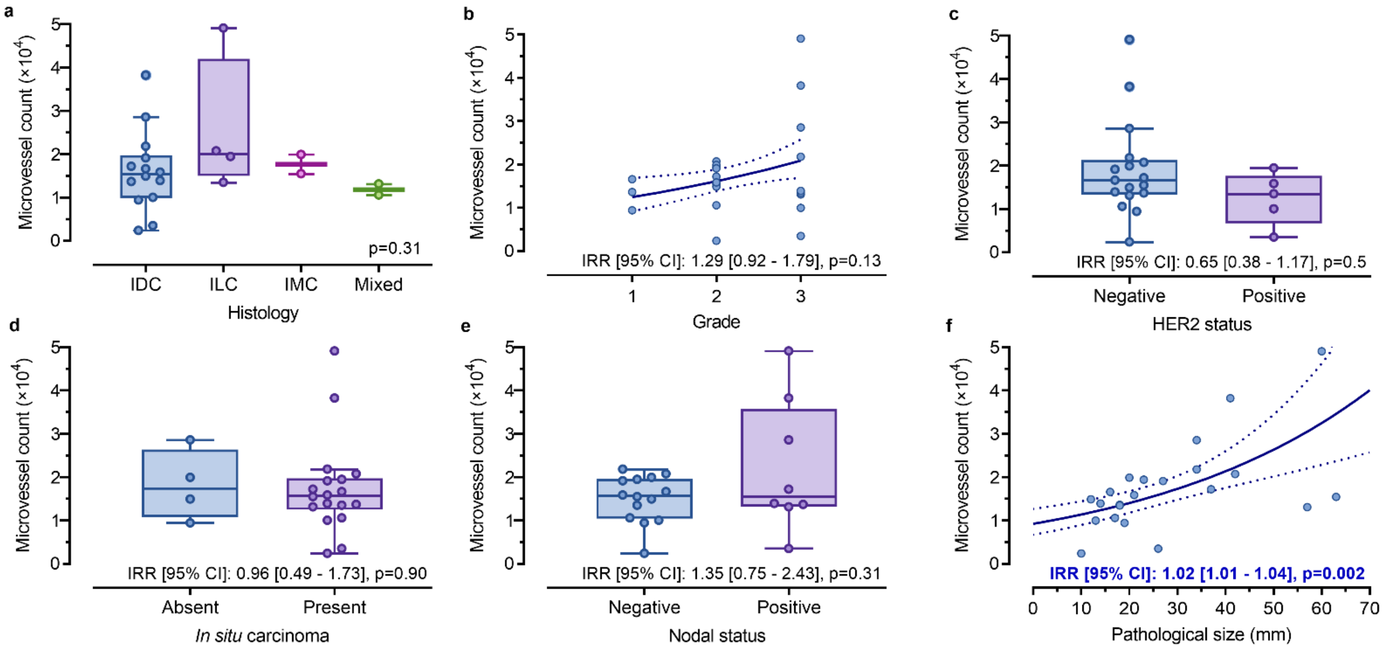

Supplemental Fig. 1 Associations between CD31 tumour microvessel count and clinicopathological variables. Analyses utilised negative binomial regression with CD31 microvessel count as the dependent variable. Regression analyses against nodal status used negative binomial mixed models with random intercepts for subjects. Effect size estimates are given as incident rate ratios (IRR) with 95% confidence intervals (CI).

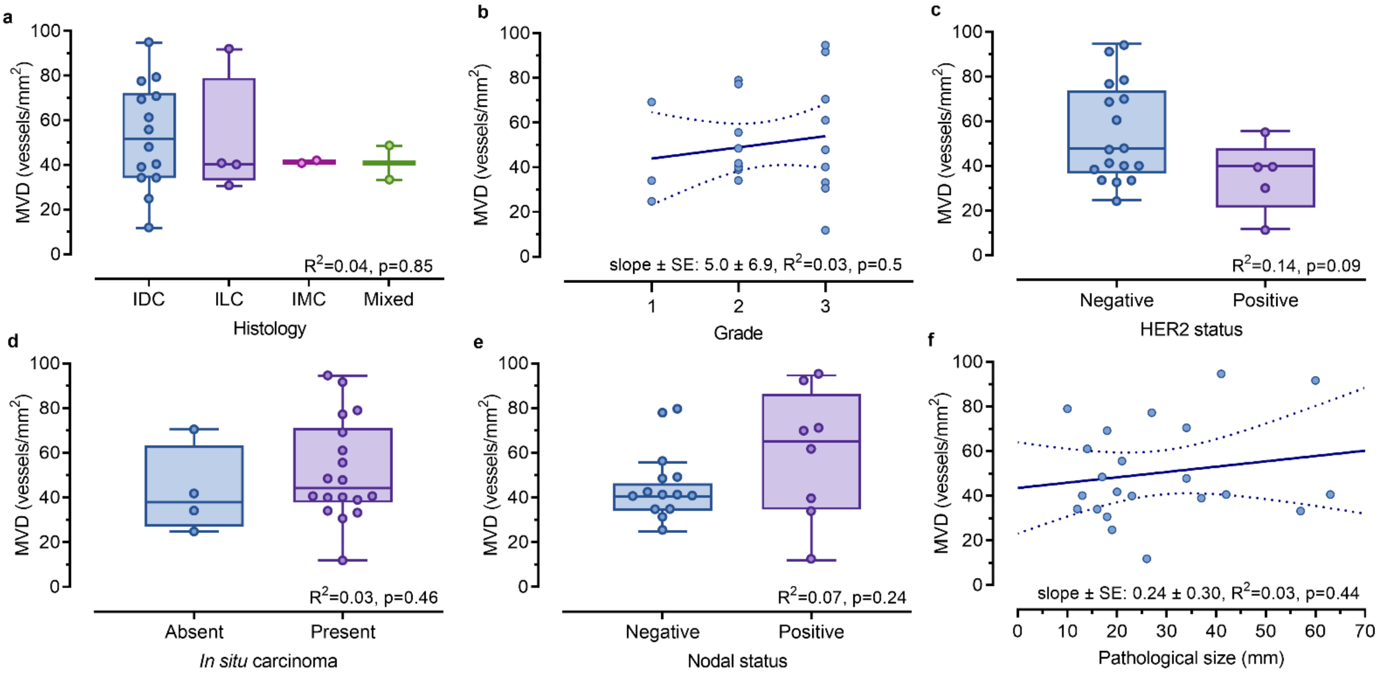

Supplemental Fig. 2 Associations between microvessel density (MVD; vessels/mm^2) and clinicopathological variables. Analyses utilised linear regression with CD31 MVD as the dependent variable. Regression analyses against nodal status used mixed models with random intercepts for subjects. Effect sizes represent R^2.

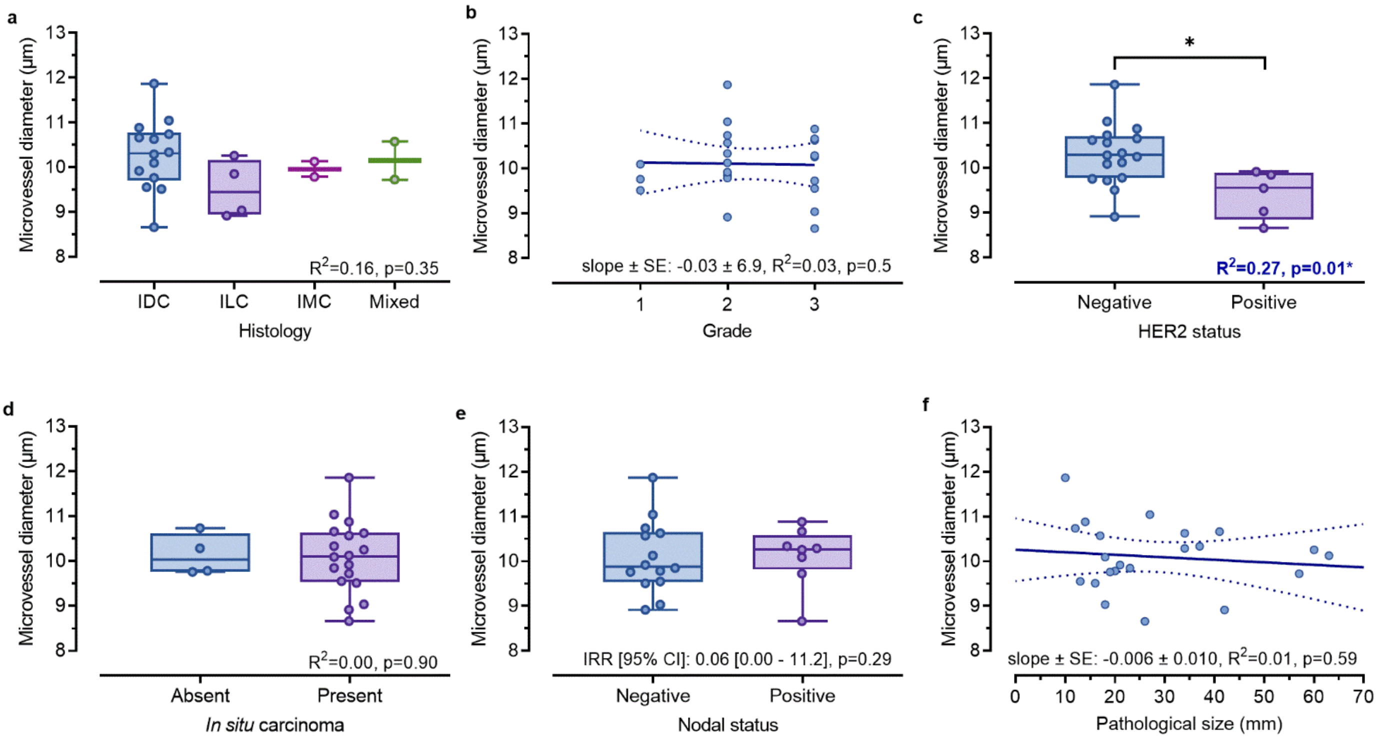

Supplemental Fig. 3 Associations between microvessel diameter (µm) and clinicopathological variables. Analyses utilised linear regression with CD31 microvessel diameter as the dependent variable. Regression analyses against nodal status used mixed models with random intercepts for subjects. Effect sizes represent R^2.

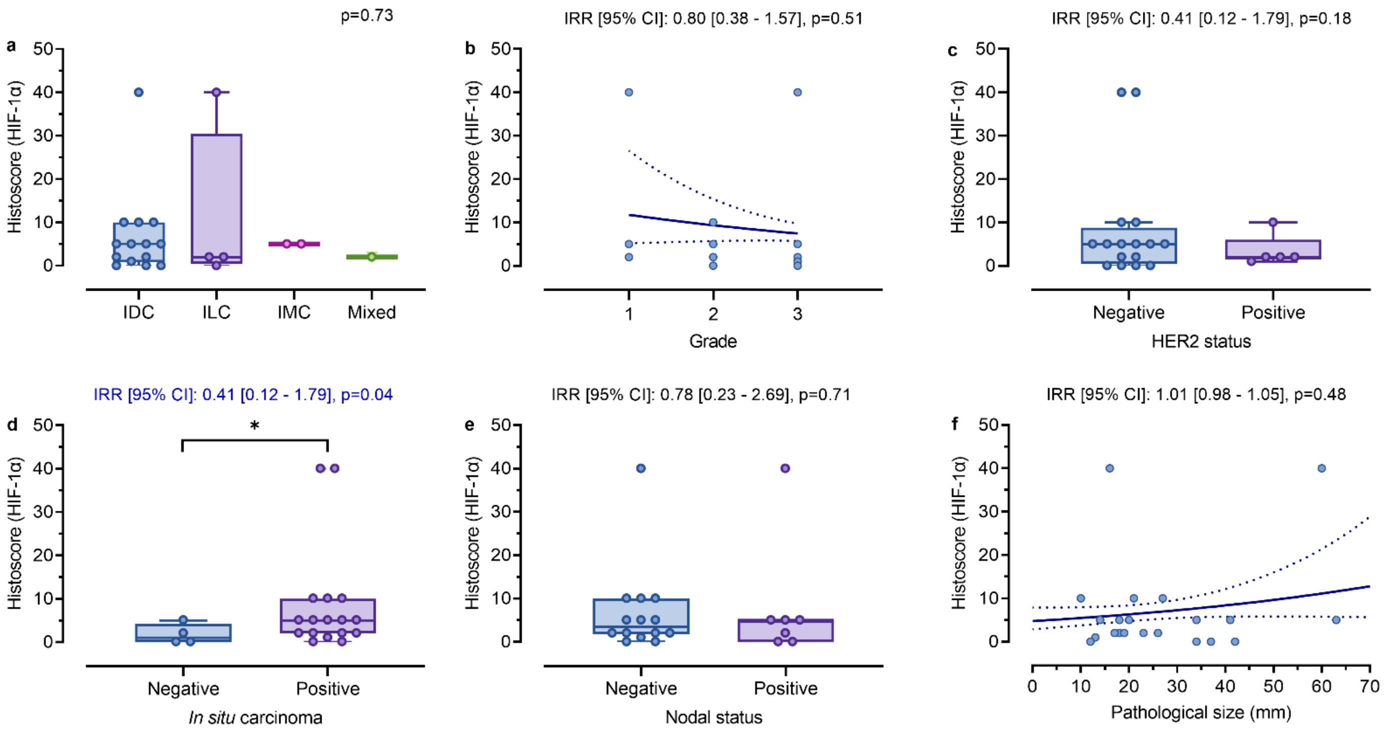

Supplemental Fig. 4 Associations between HIF-1α histoscores and clinicopathological variables. Analyses utilised negative binomial regression with HIF-1α histoscore as the dependent variable. Regression analyses against nodal status used negative binomial mixed models with random intercepts for subjects. Effect size estimates are given as incident rate ratios (IRR) with 95% confidence intervals (CI).

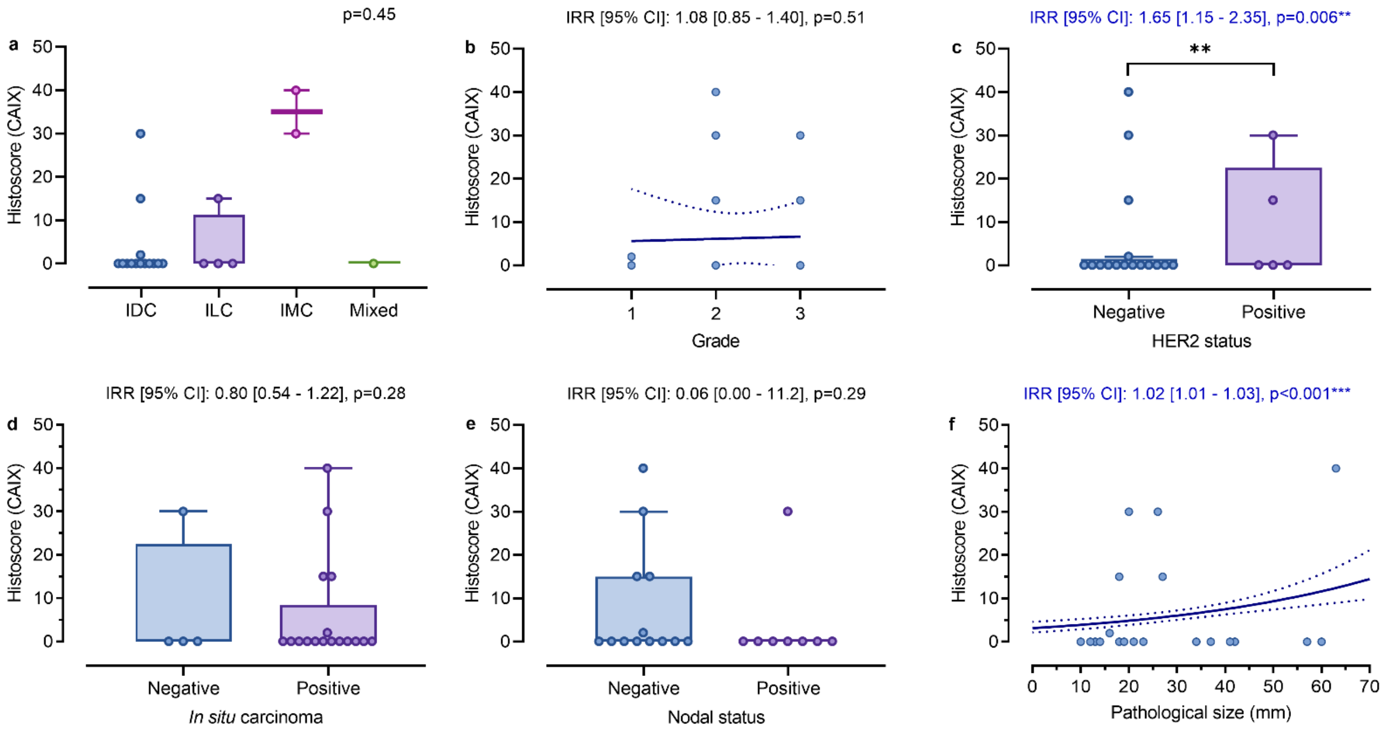

Supplemental Fig. 5 Associations between CAIX histoscores and clinicopathological variables. Analyses utilised zero-inflated negative binomial regression with CAIX histoscore as the dependent variable. Regression analyses against nodal status used zero-inflated negative binomial mixed models with random intercepts for subjects. Results are given for the negative binomial-distribution part of the model. Effect sizes are given as incident rate ratios (IRR) with 95% confidence intervals (CI). Regression models were implemented using the function gamlss from the R package GAMLSS.
